# ATTR Amyloidosis: development of cardiac symptoms during 6 years of follow up in different ATTR-variants

**DOI:** 10.1186/1750-1172-10-S1-O18

**Published:** 2015-11-02

**Authors:** Sebastiaan Klaassen, Jasper Tromp, Bouke Hazenberg, Dirk-Jan Van Veldhuisen, Maarten Van Den Berg, Peter Van Der Meer

**Affiliations:** 1 University Medical Center Groningen, Department of Cardiology, 9713AV, Groningen, The Netherlands; 2 University Medical Center Groningen, Department of Rheumatology and Clinical Immunology, 9713AV, Groningen, The Netherlands

## Background

At least 100 mutations in the TTR gene are known to cause ATTR amyloidosis. Limited information is available with regard to the progression of cardiac involvement in different amyloidogenic TTR mutations. This study investigates clinical characteristics and cardiac involvement of some ATTR-associated genotypes in the Netherlands.

## Methods

Clinical data and information with regard to survival was collected for all ATTR patients between 1994 and 2014 in the University Medical Centre Groningen, which is the Dutch national centre of expertise. In total 114 consecutive patients carrying 10 different TTR mutations were admitted. Patients were divided into different groups based on their mutation. Only mutations present in more than 5 patients were included for further analysis. The TTR mutations studied were Val71Ala (n=9), Tyr114Cys (n=21) and Val30Met (n=31). For each mutation clinical and demographical characteristics, laboratory measurements and echocardiography data were collected at baseline and follow-up. Baseline was determined as time of diagnosis based on histological confirmation of amyloid, end of follow-up was defined as the last complete measurement available. The primary endpoint was the development of heart failure (HF) at follow-up, defined as NT-proBNP plasma levels above 125 ng/L.

## Results

Patients were predominantly male (59%). Mean follow-up was 6 + 4 years and did not differ among the mutations (P=0.144). Patients with the Val71Ala and Tyr114Cys genotypes were younger at diagnosis compared to the Val30Met genotype (44 and 50 vs.55 years respectively; P=0.057). Most patients presented with neurological symptoms (79%) and most of the remaining patients presented because of familial amyloidosis (10 %). At baseline the NT-proBNP levels were raised in Val71Ala (11%), in Tyr114Cys (14%) and inVal30Met (35%). NT-proBNP levels did not differ among the mutations; median 20 (18-28), 97 (64-288) and 170 (112-365) ng/L (P= 0.096). At follow-up, NT-proBNP levels of only Val71Ala and Val30Met increased further, median increase 206 (44-221) and 194 (89-706) ng/L (P=0.043 & P<0.001). Echocardiography in patients with Val30Met showed an increase in thickness of septal wall and posterior wall, median 2 (0-4) and 3 (0-3) mm as compared to the other mutations (p=0.005 & p<0.001). The 5-year survival rates among mutations were similar (78%, 81% vs. 81%, P=0.978).

## Conclusions

During follow-up, levels of NT-proBNP in both Val71Ala and Val30Met patients increased significantly, whereas the posterior wall thickness in Val30Met also increased. These results signify that despite initial presentation with neurological symptoms, cardiac involvement is already present and progressive in some of the TTR mutations.

